# Causes of perinatal mortality and associated maternal factors in a tertiary referral hospital of Gandaki province of Nepal: a cross-sectional study from a hospital-based surveillance

**DOI:** 10.1186/s12884-022-04596-0

**Published:** 2022-03-24

**Authors:** Nuwadatta Subedi, Dipendra Kandel, Tumaya Ghale, Bandana Gurung, Bandana Shrestha, Sabita Paudel

**Affiliations:** 1 Department of Forensic Medicine, Gandaki Medical College Teaching Hospital and Research Center, Gandaki Province Pokhara, Nepal; 2 Green Pastures Hospital - International Nepal Fellowship, Gandaki Province Pokhara, Nepal; 3 Department of Anesthesiology, Gandaki Medical College Teaching Hospital and Research Center, Gandaki Province Pokhara, Nepal; 4 Department of Obstetrics and Gynecology, Gandaki Medical College Teaching Hospital and Research Center, Gandaki Province Pokhara, Nepal; 5 Department of Pediatrics, Gandaki Medical College Teaching Hospital and Research Center, Gandaki Province Pokhara, Nepal; 6 Department of Pharmacology, Gandaki Medical College Teaching Hospital and Research Center, Gandaki Province Pokhara, Nepal

**Keywords:** Antenatal care, Mortality surveillance, Perinatal mortality, Preterm labor, Stillbirth

## Abstract

**Background:**

Perinatal and neonatal death rates have decreased recently but it still poses a major challenge to the health system of Nepal. The study was conducted to explore the pattern and causes of perinatal deaths.

**Methods:**

This was a descriptive cross-sectional study conducted from September 2020 to June 2021 using the data of perinatal mortality of three years from June 15, 2017, to June 14, 2020. The demographic parameters of the mother consisted of maternal age, place of residence, ethnicity, antenatal care, the number of antenatal visits, gravida, gestational weeks, and the mode of delivery. The causes of death were categorized into fetal and early neonatal deaths. Fetal deaths were further classified as macerated stillbirth and fresh stillbirth. The attribution of the causes of deaths to fetal/neonatal and maternal conditions was done as per the guidelines of the WHO application of ICD-10 to deaths during the perinatal period.

**Results:**

There were a total of 145 perinatal deaths from 144 mothers among which 92 (63.5%) were males. Ten mothers (6.9%) had not sought even single antenatal care, whereas 32 (22.9%) had visited for antenatal care one to three times. At least one cause of death was identified in 114 (78.6%) and remained unknown in 31(21.4%) cases. Among the 28 cases of macerated stillbirths, the cause of death was not identified in 14 (50%), whereas preterm labor was attributed to the cause of death in four (14.3%). In 53 of the fresh stillbirths, intrapartum hypoxia was identified as the cause of death in 20 (37.7%) cases, preterm labor in nine (17%), and was left unknown in 15 (28.3%) cases. Among the 64 early neonatal deaths, prematurity was attributed as the cause of death in 32 (50%) cases, birth asphyxia, and infections each in 11 (17.2%).

**Conclusions:**

The perinatal mortality surveillance system identified the causes of death in most of the cases in our observation. Prematurity was identified as the commonest cause of early neonatal deaths and preterm labor was the commonest cause responsible for perinatal deaths overall. The perinatal deaths should be investigated to establish exact causes of deaths which can be useful to develop prevention strategies.

## Background

According to the World Health Organization global data, 2.4 million children died in the first month of life only in 2019 and approximately 6700 newborn deaths occur every day [1]. One in three of such deaths can be prevented largely [2]. Childbirth is often looked at as a natural process that requires no planning or medical care in different parts of developing countries. Though the conditions are improving recently, effective delivery of better health care services can further decrease the stillbirths and neonatal deaths to a great extent in low- and middle-income countries (LMICs) [3]. Most of the neonatal deaths in developing countries are cases that are delivered at home [4]. Neonatal survival can be improved by implementing community-based interventions like improvement of education, decrement in poverty, empowerment of females and overall development and efforts of continuous provision of health services [5]. Fetal death with completed pregnancy of 22 weeks and the death of all newborns within seven days of life in aggregate is known as perinatal mortality. Death within the first week of life is categorized as early neonatal mortality while the sum of stillbirth and neonatal mortality (death within the first 28 days of life) is known as extended perinatal mortality [4]. The department of Family Health Division of the Ministry of Health and Population of Nepal investigates perinatal deaths under the program, Maternal and Perinatal Death Surveillance and Response (MPDSR) System [6]. Perinatal mortality is significantly contributed by stillbirths and prematurity in Nepal, and it can be minimized by provisions for antenatal surveillance and monitoring, timely referral of high-risk pregnancies and good neonatal care to preterm neonates [7].

In Nepal, perinatal mortality in 2016 was 31 per 1000 total births and it has dropped appreciably when compared to data of 2006 (45 per 1000 total birth). Between 1996 and 2016, neonatal mortality fell from 50 to 21 deaths per 1,000 live births [8]. Although the perinatal and neonatal death rates have decreased recently [8], it still possesses a major challenge to the health system of Nepal. Because such information is scarce in the Nepalese context, quantifying such deaths and finding out their cause is the first step in acknowledging this problem and gathering baseline data. This will help in initiating effort to come up with a solution plan, to assess whether these solutions are effective, which will ultimately help in forming relevant guidelines. The study was conducted to explore the pattern and causes of perinatal deaths presented to a tertiary referral hospital of Gandaki Province of Nepal.

## Methods

This was a descriptive cross-sectional study conducted at a tertiary referral hospital of Gandaki Province of Nepal from September 2020 to June 2021 and the date of data collection was from 1 to 30 December 2020. There is a provision of recording relevant data in all perinatal deaths at the hospital and it is one of the teaching hospitals with Maternal and Perinatal Death Review (MPDR) system in function. All fetal deaths with a completed gestational age of 22 weeks and all newborns delivered after completed 22 weeks of pregnancy and died within seven days of life were included, whereas the fetal deaths of less than 22 weeks of gestational age and deaths of neonates with survival days of more than seven days were excluded from the study. The gestational age was based on the last menstrual period. All perinatal deaths registered from June 15, 2017, to June 14, 2020, that were available in the record section of GMC were collected. There was a total of 145 perinatal deaths, and all were included in our analysis.

The data were collected from the records available at the record section of GMC as a record review. Structured proforma was designed to collect data on demographic parameters of the mother and the fetus or neonate. The demographic parameters of the mother consisted of maternal age, place of residence (rural or urban), ethnicity, antenatal care, the number of antenatal visits, gravida, gestational weeks, and the mode of delivery. The causes of death were categorized into fetal and early neonatal deaths. Fetal deaths were further classified as macerated stillbirth and fresh stillbirth. The non-specific causes recorded in the cause of death, for instance, cardiopulmonary arrest without any underlying cause were disregarded. The cause of death also included the maternal conditions which could have been responsible to cause the death of the fetus/neonate. All the recorded causes of death were entered and categorized. The attribution of the causes to deaths to fetal/neonatal and maternal conditions was done as per the guidelines of the WHO application of ICD-10 to deaths during the perinatal period: ICD-PM [9]. The maternal causes contributing to the death of the fetus/neonate were classified into five groups based on the classification of maternal conditions in ICD-PM [9].

The data were entered in Microsoft Excel and further analyses were done by SPSS 25.0 to calculate frequency, mean and standard deviation. Ethical approval was obtained from the Institutional Review Committee of Gandaki Medical College (Ref. No 98/77/78, dated November 10, 2020).

## Results

There was a total of 145 perinatal deaths from 144 mothers registered at the record section of Gandaki Medical College in the study duration. In one case of delivery, both the neonates had died. A total of 92 (63.5%) of the babies were males. Most of the mothers had an age group of 20 to 34 years with 107 (74.3%) cases in total. The distribution of place of residence rural and urban was almost similar and most of the participants (43, 29.9%) were Janajati. Ten (6.9%) mothers had not sought even single antenatal care, whereas 32 (22.9%) had visited for antenatal care one to three times. The demographic distribution of the study population for fetal and early neonatal deaths is demonstrated in Table 1.

**Table 1:** Demographic profile of study population

**Variables**		**Type of death**		**Total (*N* = 145 for fetus/neonates), (*N* = 144 for mothers)** **n (%)**
		**Fetal (*n* = 81)** **n (%)**	**Early neonatal (*n* = 64 for neonates, *n* = 63 for mothers)** **n (%)**	
**Maternal age (years)**	< 20	22 (27.2)	8 (12.7)	30 (20.8)
	20–34	55 (67.9)	52 (82.5)	107 (74.3)
	> 34	4 (4.9)	3 (4.8)	7 (4.9)
**Place of residence**	Rural	39 (48.1)	30 (47.6)	69 (47.9)
	Urban	42 (51.9)	33 (52.4)	75 (52.1)
**Ethnicity**	Brahmin	21 (25.9)	16 (25.4)	37 (25.7)
	Chhetri	16 (19.7)	11 (17.5)	27 (18.7)
	Dalit	19 (23.5)	15 (23.8)	34 (23.6)
	Janajati	22 (27.2)	21 (33.3)	43 (29.9)
	Muslim	3 (3.7)	0 (0)	3 (2.1)
**Antenatal care** **(Number of times)**	0	5 (6.2)	5 (7.9)	10 (6.9)
	1–3	16 (19.7)	17 (27.0)	33 (22.9)
	> 3	60 (74.1)	41 (65.1)	101 (70.1)
**Type of pregnancy**	Single	79 (97.5)	56 (88.9)	135 (93.7)
	Twin	2 (2.5)	7 (11.1)	9 (6.3)
**Gravida**	1	36 (44.4)	28 (44.4)	64 (44.4)
	2	22 (27.2)	24 (38.1)	46 (31.9)
	> 3	23 (28.4)	11 (17.5)	34 (23.6)
**Gestational weeks**	< 34	39 (48.1)	32 (50.0)	71 (49.3)
	34–37	14 (17.3)	9 (14.3)	23 (15.6)
	> 37	28 (34.6)	22 (34.9)	50 (34.7)
**Sex of the foetus/neonate**	Male	49 (60.5)	43 (67.2)	92 (63.5)
	Female	32 (39.5)	21 (32.8)	53 (36.5)

Number of foetus/neonates: 145, Number of mothers: 144 (in one case, both the neonates died), and percentage in "total" is calculated accordingly. The percentage is calculated for the variables in each type of death as well

The obstetric condition of the mother at the time of presentation to the hospital and mode of delivery is shown in Table 2. Most of the mothers (67, 46.5%) were not in labor and 32 (22.2%) were brought in the active phase of labor. A total of 124 (86.1%) had undergone vaginal delivery, which includes one case of forceps delivery and vacuum delivery each and 20 (13.9%) were delivered by caesarian section.

**Table 2:** Obstetric condition of mother at time of presentation and mode of delivery

**Variables**		**Fetal deaths (*n* = 81)** **n (%)**	**Early neonatal deaths (*n* = 63)** **n (%)**	**Total (*N* = 144),** **n (%)**
**Obstetric condition of mother**	Not in labour	48 (59.3)	19 (30.2)	67 (46.5)
	Active phase of labour	17 (21.0)	15 (23.8)	32 (22.2)
	Latent phase of labour	15 (18.5)	11 (17.5)	26 (18.1)
	Post-partum	0 (0)	14 (22.2)	14 (9.7)
	Third stage of labour	1 (1.2)	5 (7.9)	6 (4.2)
**Mode of delivery**	Vaginal	75 (92.6)	49 (77.8)	124 (86.1)
	Caesarian Section	6 (7.4)	14 (22.2)	20 (13.9)

Among the total 145 perinatal deaths, at least one cause of death was identified in 114 (78.6%) and unknown in 31(21.4%) cases. The causes of deaths and the category according to the macerated stillbirths, fresh stillbirths, and early deaths are presented in Table 3. Among the identified 28 cases of macerated stillbirths of the total 145 cases studied, the cause of death was not identified in 14 (50%) of the cases whereas preterm labor was attributed to the cause of death in four (14.3%), congenital anomalies in three (10.7%) cases. In the case of 53 of the fresh stillbirths, intrapartum hypoxia was identified as the cause of death in 20 (37.7%) cases whereas preterm labor was the cause in nine (17%), and it was left unknown in 15 (28.3%) cases. Among the 64 early neonatal deaths, prematurity was attributed as the cause of death in 32 (50%) cases, birth asphyxia, and infections each in 11 (17.2%) with only two (3.1%) cases where the cause of death was not identified.

**Table 3:** Causes of fetal and early neonatal deaths

Cause of death	Frequency	Percent
Causes of macerated stillbirths (*n* = 28)		
Preterm labor	4	14.3
Congenital anomalies	3	10.7
Antepartum hypoxia	2	7.1
Hypertensive disorder	2	7.1
Cord around neck	1	3.6
Placental deficiency	1	3.6
Twin-to-twin transfusion syndrome	1	3.6
Unknown	14	50.0
Causes of fresh stillbirths (*n* = 53)		
Intrapartum hypoxia	20	37.7
Preterm labor	9	17.0
Hypertensive disorders	3	5.7
Congenital anomalies	3	5.7
Placenta abruption	2	3.8
Infections	2	3.8
Intrauterine growth retardation	1	1.9
Cord around neck	1	1.9
Meconium aspiration syndrome	1	1.9
Oligohydramnios	1	1.9
Unknown	15	28.3
Causes of early neonatal deaths (*n* = 64)		
Prematurity	32	50.0
Birth Asphyxia	11	17.2
Infections	11	17.2
Respiratory distress syndrome	7	10.9
Meconium aspiration syndrome	5	7.8
Congenital anomalies	4	6.2
Aspiration pneumonia	2	3.1
Disseminated Intravascular coagulation	1	1.6
Hypoxic ischemic encephalopathy	1	1.6
Intracranial bleed	1	1.6
Neonatal hypoglycemia	1	1.6
Rh Incompatibility	1	1.6
Unknown	2	3.1

Multiple causes of death were included

From the records available, we picked up the maternal causes identified as a contributor to the perinatal deaths. In 56 (38.9%) of the total 144 cases, some maternal causes were identified in which preterm labor as the complications of labor and delivery was the commonest cause in 35 (25.3%) followed by the maternal medical and surgical conditions in 13 (9%) cases. The other maternal factors and their category according to the ICD-PM are presented in Table 4.

**Table 4:** Maternal causes identified in perinatal deaths

ICD-PM maternal condition group	Maternal factors	Fetal deaths (*n* = 81) Frequency (%)	Early neonatal deaths (*n* = 63) Frequency (%)	Total (*N* = 144) Frequency (%)
**M1: Complications of placenta, cord and membranes (*n* = 8)**	Antepartum hemorrhage	4 (4.9)	1 (1.6)	5 (3.5)
	Cord around neck	2 (2.5)	0 (0)	2 (1.4)
	Placental deficiency	1 (1.2)	0 (0)	1 (0.7)
**M2: Maternal complications of pregnancy (*n* = 2)**	Oligohydramnios	1 (1.2)	0 (0)	1 (0.7)
	Multiple pregnancy	1 (1.2)	0 (0)	1 (0.7)
**M3: Other complications of labour and delivery (*n* = 35)**	Preterm labour	10 (12.3)	25 (39.7)	35 (25.3)
**M4: Maternal medical and surgical conditions (*n* = 13)**	Hypertensive disorder	7 (8.6)	2 (3.2)	9 (6.2)
	Sepsis	0 (0)	4 (6.3)	4 (2.8)
	Preeclampsia	1 (1.2)	1 (1.6)	2 (1.4)
**M5: No maternal condition (*n* = 88)**		54 (66.7)	34 (54.0)	88 (61.1)

## Discussion

We have analyzed the data of perinatal death review from a tertiary referral center in  Gandaki province Nepal and found that at least one cause of death was identified in 78.6% cases with intrapartum hypoxia as the commonest cause in fresh stillbirths and prematurity among the early neonatal deaths whereas it remained unexplored in half of the macerated stillbirths. Perinatal deaths affect the dynamics of the parents and family and their social wellbeing. The parents regard that society underestimates their loss; therefore, the community leaders and health care providers should pay attention to the family members who have suffered a perinatal death [10]. Along with its effects on the family members, perinatal death also creates emotional effects on obstetricians and physicians [11]. Infant-mortality rates have been declining in many low and middle-income countries [12], but still, it poses challenges in some localities in developing countries. The mechanism of counting and accounting for deaths in a systematic manner is important and it can provide evidence to determine changes in clinical practice and to develop guidelines and training packages for preventive measures as well [13].

The identification of the causes of perinatal deaths, from both the maternal and the perinatal side, is important to design and develop preventative and therapeutic measures. The certification of the cause of perinatal deaths is not complete without identifying the maternal conditions leading to the insult to the child [9]. We have presented the maternal conditions in our analysis that could have contributed to the death of the child.

The cause of death could not be identified in more than one-third of stillbirths in our study. It is almost similar to the observation from a study in East Timor [14]. The establishment of the cause of death in stillbirths can be challenging because of the unavailability of investigations and the non-performance of an autopsy. To overcome this, a complete diagnostic autopsy (CDA) should be conducted. But there are several constraints to CDA including cultural and religious beliefs, prolonging the time for the funeral, and lack of resources and trained manpower. Recently, minimally invasive tissue sampling (MITS) has been used successfully in different LMICs to explore the cause of death in different age groups and it has proven more useful in perinatal and child deaths [15, 16]. In Nepal, CDA is not conducted unless there is a medicolegal necessity and thus proper cause of death is missed in many cases. As establishing the cause of death is important for prevention and public health planning proposes, the MPDSR program is better supplemented with MITS. MITS is a relatively non-invasive procedure, can be conducted with trained technicians or pathologists in health facilities and even in the community, it is feasible in our context and can prove very useful to establish the cause of death in perinatal deaths [16]. Evidence has shown MITS as an acceptable procedure in LMICs with different cultures and religions, as it is less disfiguring, the procedure takes less time than CDA, thus causes fewer delays for funerals [17, 18]. A consent rate of 71% has been presented from a study involving under-5 mortality and stillbirths, at five sites in Mozambique, South Africa, Kenya, Mali, and Bangladesh [19]. Verbal autopsy can be another tool for identifying the causes of death particularly in the resource limited settings where many deaths occur outside the hospital facilities and certification of exact causes of death is usually impossible. In fact, verbal autopsy is a partial solution to identify the causes of death in the absence of clinical investigations and CDA. A verbal autopsy is a procedure to determine the cause of death based on a structured conversation with the deceased's relatives or caregivers who had sufficient information about the conditions of the deceased. This is performed using a standardized questionnaire that explores the medical information, symptoms, signs, and circumstances that lead to death. The WHO has standardized the verbal autopsy tool to maintain the standards of ascertaining the causes of death [20] and the tool has been proven to identify main causes of neonatal deaths in India [21] and Pakistan [22].

Prematurity was identified as the cause of death in half of the cases of early neonatal deaths. This could be attributed to the inadequacy of nutrition, lack of adequate facilities for neonatal care. Birth asphyxia and infections were the next common cause of early neonatal deaths. Infections as the cause of death are in decreasing trend in developing countries [23], though it still poses a threat if not properly addressed. In our context, the identification of specific pathogens as the causative agent of infections is often challenging due to the unavailability of specific tests. Several causes leading to neonatal deaths can be prevented if health facilities are adequately empowered.

Congenital anomalies were attributed to the cause of death in seven percent of perinatal deaths in our observation. The congenital anomalies can be prevented by proper perinatal checkups and anomaly scans. The primary prevention plan is based on public education about preconception and prenatal risks. Prevention based on reproductive options includes information services regarding teratogens and prenatal examinations for fetal abnormalities [24].

In contrast to a study utilizing verbal autopsy to identify the causes of stillbirth and neonatal deaths in the Dhanusha district of Nepal, where obstetric complications were the leading cause of mortality [12], our study found no maternal conditions in 61.1%. This could be attributed to decreasing trend of maternal factors due to the safe motherhood program conducted by the government of Nepal [25]. In our observation, preterm labor was the commonest maternal condition identified responsible for perinatal deaths. In the United States also, spontaneous preterm delivery is the leading cause of neonatal morbidity and the most common cause of hospitalization during pregnancy [26]. Preterm labor needs to be prevented and managed on time using the updated protocols to prevent foreseeable complications [27]. The identification of the maternal causes overall is important to plan the preventive measures and strategies, and this should not be neglected.

Skilled birth attendance and institutional delivery are the two essential components to reduce perinatal as well as maternal mortality. After the initiation of the program by the government of Nepal which incentivized institutional deliveries [25], maternal as well as perinatal deaths were reduced to a great extent than previous years [28]. But still, as presented by this study, the death rate was not less even though institutional delivery was practiced. The authorized bodies need to collaborate for navigating the causes of mortality so that proper guidelines could be formulated to save precious lives.

The analyses of the data obtained from the National Surveillance system was the strength of our study as it provided a clear relevant picture of perinatal mortality representing the Gandaki Province of Nepal. Our inferences will certainly be a milestone for the government to plan its programs to decrease perinatal mortality by addressing the various causative factors. Many perinatal deaths occurring at home are not reported at the hospitals so, the hospital-based surveillance might miss out such cases, which could be a limitation of our study.

## Conclusion

The perinatal mortality surveillance system identified the causes of death in most of the cases in our observation. Among the perinatal deaths reported, stillbirths were more common. The cause of death was left unknown in the majority of cases of stillbirths and the most common identified cause was intrapartum hypoxia followed by preterm labor. Prematurity was identified as the commonest cause of early neonatal deaths and preterm labor was the commonest cause responsible for perinatal deaths overall. The perinatal deaths should be investigated to establish exact causes of deaths which can be useful to develop prevention strategies.

## Data Availability

The datasets used and/or analyzed during the current study are available from the corresponding author on reasonable request.
